# A calibrated database of kinematics and EMG of the forearm and hand during activities of daily living

**DOI:** 10.1038/s41597-019-0285-1

**Published:** 2019-11-11

**Authors:** Néstor J. Jarque-Bou, Margarita Vergara, Joaquín L. Sancho-Bru, Verónica Gracia-Ibáñez, Alba Roda-Sales

**Affiliations:** Department of Mechanical Engineering and Construction, Universitat Jaume I, Castellón, Spain

**Keywords:** Electromyography - EMG, Skeleton, Muscle

## Abstract

Linking hand kinematics and forearm muscle activity is a challenging and crucial problem for several domains, such as prosthetics, 3D modelling or rehabilitation. To advance in this relationship between hand kinematics and muscle activity, synchronised and well-defined data are needed. However, currently available datasets are scarce, and the presented tasks and data are often limited. This paper presents the KIN-MUS UJI Dataset that contains 572 recordings with anatomical angles and forearm muscle activity of 22 subjects while performing 26 representative activities of daily living. This dataset is, to our knowledge, the biggest currently available hand kinematics and muscle activity dataset to focus on goal-oriented actions. Data were recorded using a CyberGlove instrumented glove and surface EMG electrodes, both properly synchronised. Eighteen hand anatomical angles were obtained from the glove sensors by a validated calibration procedure. Surface EMG activity was recorded from seven representative forearm areas. The statistics verified that data were not affected by the experimental procedures and were similar to the data acquired under real-life conditions.

## Abstract (summary)

**Table:** 

Measurement(s)	kinematics • Electromyography
Technology Type(s)	Sensor Device • electromyography
Factor Type(s)	activity of daily living
Sample Characteristic - Organism	Homo sapiens

Machine-accessible metadata file describing the reported data: 10.6084/m9.figshare.10075355

## Background & Summary

The hand is a complex functional limb with more than 20 joints controlled by more than 30 muscles that allow a wide range of activities to be performed very precisely. Knowing how complex hand movements are produced and controlled may be useful for several applications like improving prosthetic control^1^, developing realistic biomechanical hand models^2^, or improving hand rehabilitation by more adapted physiotherapy^3–5^. For this goal, synchronised kinematic and electromyographic (EMG) data are needed. Some works have attempted to link hand kinematics with forearm muscle activity^1,6–8^, but have focused on analysing specific muscles to perform very specific, simple and controlled activities and, therefore, lack the representativeness of the activities of daily living (ADL).

Although some hand kinematics datasets (performing different grasps and hand movements) are available in the literature, as well as forearm EMG datasets (usually performing hand gestures or free hand movements), very few datasets exist with simultaneously recorded kinematics and EMG^9–11^, and they have their weaknesses:

- Tasks lack representativeness of ADL: only grasping movement or static finger/hand postures recorded^9–11^.
- Motion capture system used: Kinematic data are barely reliable in some cases because of the motion capture system used^11^.
- Type of kinematic data presented: Only raw kinematic data are provided in some cases^9^ instead of properly obtained anatomical angles.
- EMG electrodes location: No indication of the exact location of electrodes in some cases^9,11^.

Most methods followed to measure hand movements fail to capture kinematics while performing ADL. Instrumented gloves seem the most effective method to collect data from all hand joints continuously without occluding problems, and with no special environmental constraints^12,13^. Note that some sensors have non-linear relationships with anatomical angles due to either their position or the influence of other joint movements^14^, and require using calibration procedures to obtain reliable angles, like that described in a previous work^15^, with a mean precision error of 4.45 degrees.

EMG is commonly used to measure muscular activity. Intramuscular (iEMG) electrodes are ideal for deep muscles, but have some disadvantages^16^: placement requires thorough knowledge of the musculoskeletal anatomy and is invasive (needle inserted into muscles) and painful. Although surface EMG (sEMG) is non-invasive and easy to apply, the recorded sEMG signals are dependent on accurate placement of electrodes over muscles^16^. Indeed a previous work^17^ has identified the most representative forearm areas (from easily identifiable landmarks) to improve sEMG electrodes placement for ADL performance in EMG activity terms.

Defining a representative set of ADL is not evident. Clinical tests, like the Sollerman Hand Function test (SHFT)^18^, are often used to evaluate the upper extremity’s functional recovery by performing tasks that simulate ADL. A recent work (Peters *et al*., 2018) suggests that studying hand kinematics and EMG data while these clinical trials are being carried out can provide a better and necessary understanding of muscle recruitment and coordination for functional recovery. Based on this suggestion, we propose considering the actions used in clinical tests to study how hand movements are produced and controlled while performing ADL.

The presented KIN-MUS UJI dataset^19^ aims to allow worldwide research groups to study the relationship between hand kinematics and muscle activity required to perform ADL. The main contribution of this dataset, compared to others, is the functional activities performed. The kinematic data are standardised as they are presented as anatomical angles following the International Society of Biomechanics (ISB) sign criteria^20^. Muscle activity is obtained from seven representative spot areas during ADL. The dataset takes a Matlab data structure (.*mat*) with kinematic data and sEMG data. These linked data are expected to foster progress in many scientific domains, such as medicine, neuroscience, rehabilitation, physiotherapy, prosthetics and computer-aided model design, and to lead, for instance, to a better understanding of human hand movements, improved rehabilitation protocols, prosthetics that better correspond to human hand behaviour and more realistic 3D biomechanical models.

## Methods

### Study participants

Twenty-two right-handed subjects (12 males, 10 females) participated in the experiment, whose mean age was 35 ± 9 years. The criteria used to select subjects were gender parity in the overall data, being aged between 20 and 65 years, and no reported upper limb pathologies. Before the experiments, all the participants gave their written informed consent. All the experiments were run in accordance with the Ethics Committee of the Universitat Jaume I.

### Acquisition protocol

#### Kinematics acquisition

**Fig. 1:**
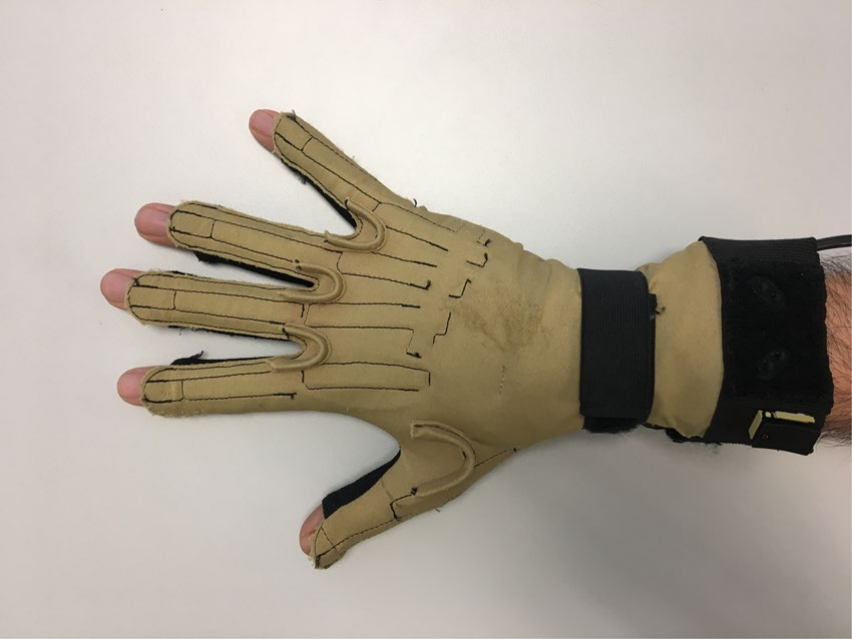
Cyberglove I device and the strap used for the wrist sensors.

The kinematic data of the right hand were acquired using a CyberGlove (CyberGlove Systems LLC) instrumented glove (Fig. 1) connected to a laptop at 100 Hz. This glove has 18 strain gauges that allow the anatomical angles of the underlying joints to be determined. All the experiments were video-recorded so as to be able to check the performance of tasks when subsequently required for data validation. Videos are not included to ensure the subjects’ privacy. To correct previous irregularities observed in the wrist sensors, a strap is placed surrounding the wrist (Fig. 1).

#### EMG acquisition

**Fig. 2:**
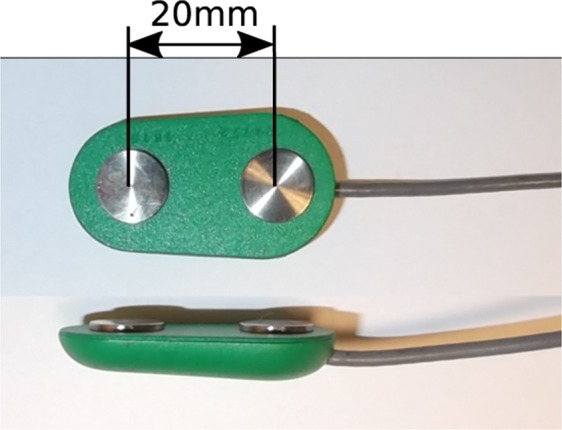
sEMG Electrodes (SX230).

**Fig. 3:**
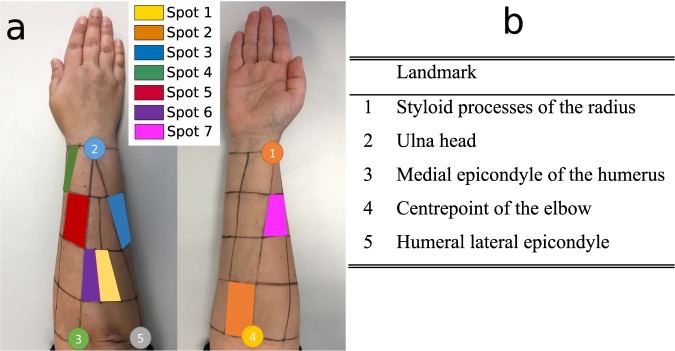
Spot areas selected for the EMG recordings and 5 anatomical landmarks used to draw the grid.

Muscle activity was recorded with an 8-channel sEMG Biometrics Ltd device at a sampling frequency of 1000 Hz. Integral dry reusable sEMG Electrodes (SX230) were used, with a gain of 1000, a bandwidth between 20 Hz–460 Hz and noise below 5 µV (Fig. 2). Electrodes were placed in the centre of the seven most representative spot areas of the right forearm (Fig. 3a), according to a previous work^17^, and were set out in a longitudinal direction. To locate these seven spot areas, a grid defining 30 spots was drawn on the subject’s forearm by using five easily identifiable anatomical landmarks (Fig. 3b), while the subject sat comfortably with an elbow resting on a table, arm flexed 90° compared to the forearm, and the palm of the hand facing the subject, as detailed in a previous work^17^. Note that these seven spots are representative of all available muscle activity of the whole forearm, because in this previous work, the entire forearm was covered without looking for any specific muscle, but trying to record all available muscle activity under the whole forearm area. Before placing electrodes, hair was removed by shaving, and skin was cleaned with alcohol.

#### Environment & tasks

**Fig. 4:**
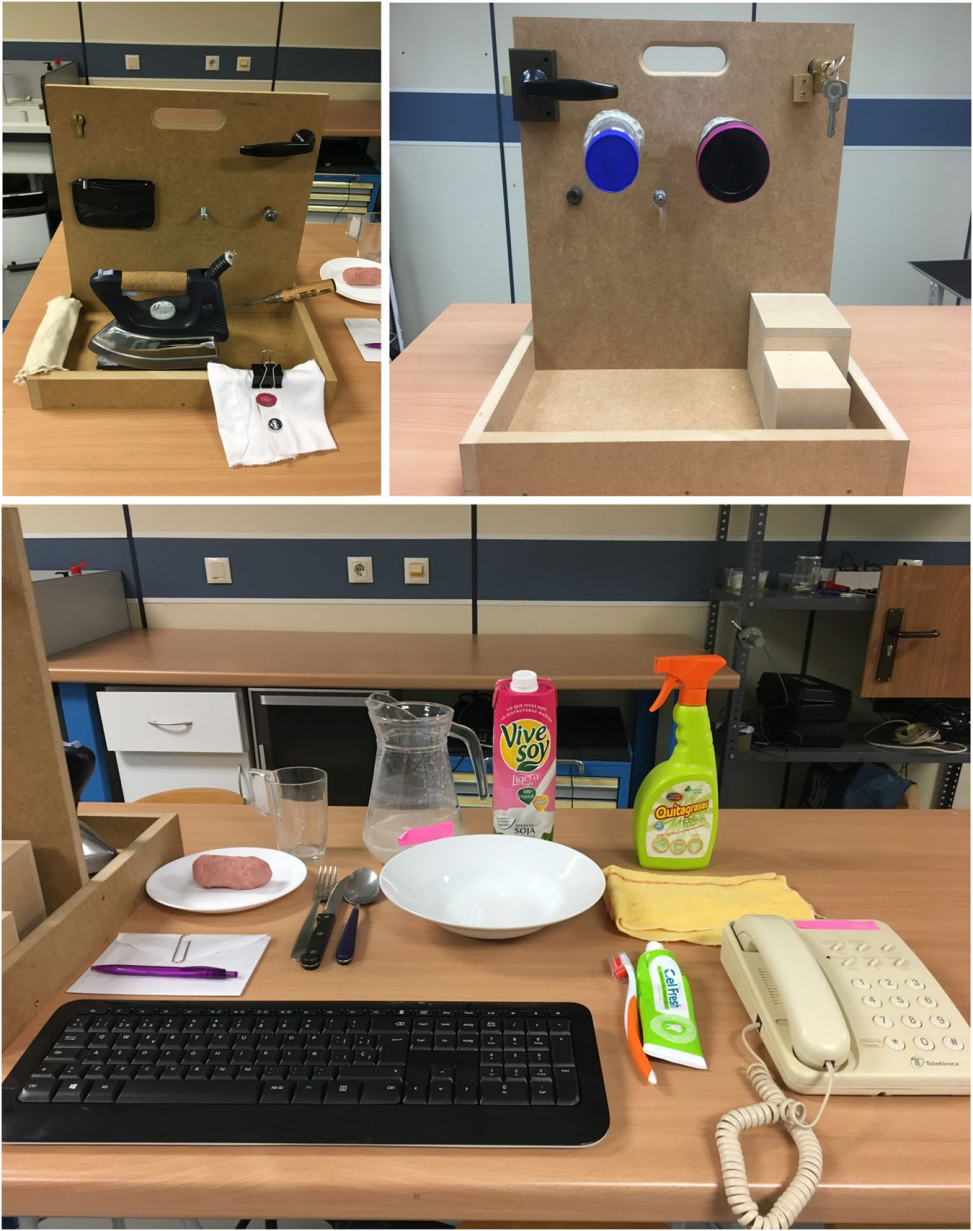
Scenario used during the experiment.

Tasks were run in a laboratory using a typical SHFT scenario. Figure 4 shows the scenario with the objects used in ADL. Tasks consisted of 26 simulated ADL, 20 of which are included in SHFT. Some ADL from SHFT were adapted to ensure their repeatability, and six further activities (A10, A15, A19, A24, A25, and A26) were added (Table 1) based on the percentage of using the commonest grasps during ADL^21^. Table 1 provides a description of each performed ADL. Some recordings were performed with the subject standing and others while they sat on a chair (as specified in Table 1). The participants were given clear instructions as to how to perform each task, including details like the angle of rotation of the key (A8), the position of the coin (A1 & A3), the angle of rotation of the door handle (A9) or the amount of water to be poured (A21). Subjects were told to start and end each task in the same posture: arms relaxed on each side of their body, when the subject was standing, or arms resting in a relaxed position on a table when sitting. The subjects could practice each task as many times as necessary in advance to become familiar with its performance before recordings. While carrying out each task, the operator marked (or labelled) the time stamp of two specific events (using the specific EMG/glove software), which were later used to separate different phases or actions: when any part of the hand came into contact with the object and when the hand released the object, to separate reaching/releasing periods from the manipulation ones. Video records of two activities (#4 and #19) are provided to have a better idea of the task performance (recording apparatus and task phases).

**Table 1:** Description of the ADL performed.

ADLs	Description
A1	Collecting a coin and putting it into a change purse
A2	Opening and closing a zip
A3	Removing the coin from the change purse and leaving it on the table
A4	Catching and moving two different sized wooden cubes
A5	Lifting and moving an iron from one marked point to another
A6	Taking a screwdriver and turning a screw clockwise 360° with it
A7	Taking a nut and turning it until completely inserted inside the bolt
A8	Taking a key, placing it in a lock and turning it counter-clockwise 180°
A9	Turning a door handle 30°
A10	Tying a shoelace
A11	Unscrewing two lids and leaving them on the table
A12	Passing two buttons through their respective buttonhole using both hands
A13	Taking a bandage and putting it on his/her left arm up to the elbow
A14	Taking a knife with the right hand and a fork with the left hand and splitting a piece of clay (sitting)
A15	Taking a spoon with the right hand and using it 5 times to eat soup (sitting)
A16	Picking up a pen from the table, writing his/her name and putting the pen back on the table (sitting)
A17	Folding a piece of paper with both hands, placing it into an envelope and leaving it on the table (sitting)
A18	Taking a clip and putting it on the flap of the envelope (sitting)
A19	Writing with the keypad (sitting)
A20	Picking up the phone, placing it to his/her ear and hanging up the phone (sitting)
A21	Pouring 1L of water from a carton into a jug (sitting)
A22	Pouring water from the jug into the cup up to a marked point (sitting)
A23	Pouring the water from the cup back into the jug (sitting)
A24	Putting toothpaste on the toothbrush
A25	Using a spray over the table 5 times
A26	Cleaning the table with a cloth for 5 seconds

A reference posture (hands resting flat on a table with fingers and thumbs close together, and middle fingers aligned with forearms) was recorded before recording the hand kinematics during the selected ADL, and was considered zero for all the rotation angles. Seven records of maximum voluntary contraction (MVC) were made: flexion and extension of the wrist, flexion and extension of fingers, pronation of the forearm, ulnar deviation of the wrist, and elbow flexion. By taking a comfortable posture, the subjects were asked to exert maximum effort without the help of other muscles than those of the forearm and hand. The order followed by each subject during recordings was: firstly, the seven MVC were performed by repeating each MVC 3 consecutive times and resting for 3 minutes between each repetition. Then the reference posture followed by each ADL was recorded in ascending order (from 1 to 26).

### Kinematic signal processing

#### Calculating angles

**Fig. 5:**
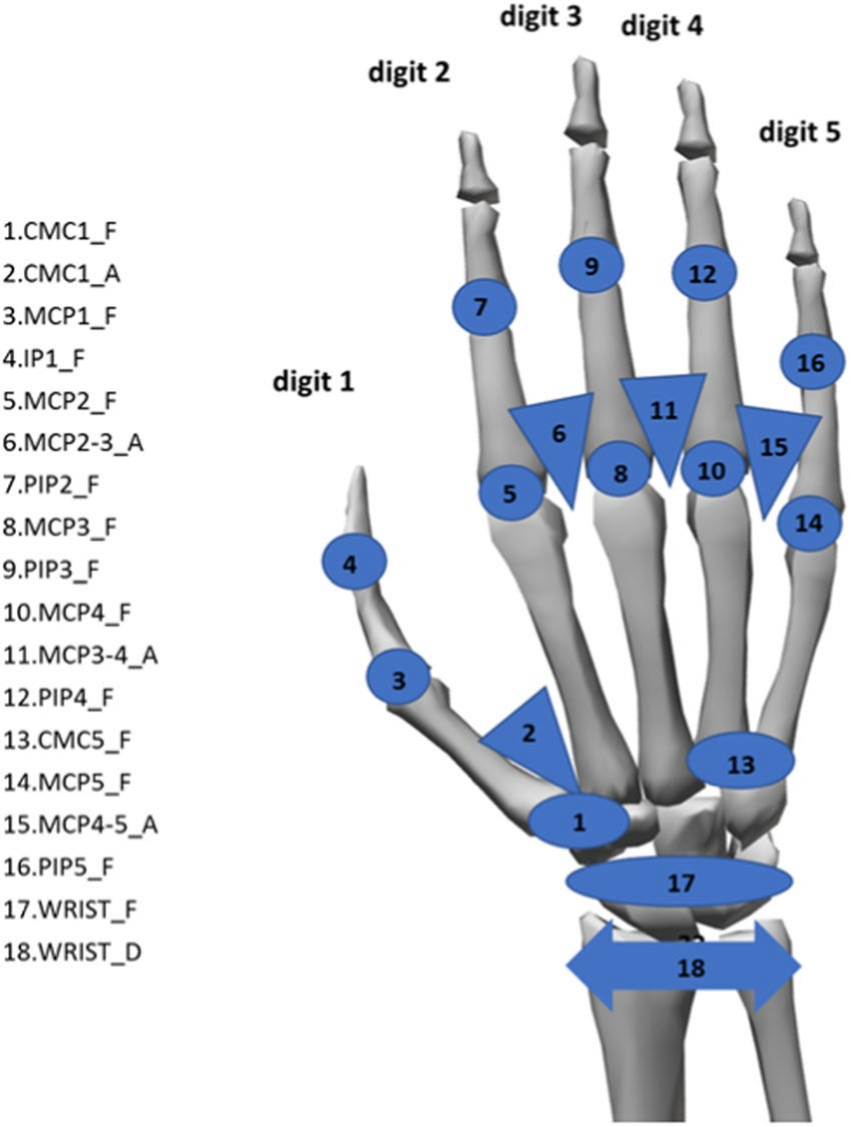
List of recorded anatomical angles. Nomenclature: _F for flexion (circles or ellipses), _A for abduction (triangles), _D for deviation (double arrow); 1 to 5, digits. Joints: IP for interphalangeal joints, PIP for proximal interphalangeal joints, MCP for metacarpophalangeal joints, CMC for carpometacarpal joints, CMC5_F for palmar arch.; WRIST for wrist joint.

The joint angles rotated from the reference posture were computed by transforming the raw data (which is available upon request) obtained from the glove sensors according to a non-linear calibration protocol proposed in previous works^15^. This protocol includes determining gains and also some corrections because of cross-coupling effects for specific anatomical angles. The list of anatomical angles obtained according to the protocol is shown in Fig. 5.

#### Filtering

The kinematic data were filtered with a second-order two-way low-pass Butterworth filter with a cut-off frequency of 5 Hz.

### EMG signal processing

#### Calculating muscle activity

Muscle activity was computed through normalisation of sEMG records by dividing them by the maximal values from any record (7 MVCs and 26 ADL) measured for each subject, in order to allow comparison of sEMG activity from the same spot of different individuals or to compare sEMG activity between different spots.

#### Filtering

The sEMG records were filtered with a fourth-order bandpass filter between 25–500 Hz, rectified, filtered by a fourth-order low-pass filter at 8 Hz, and smoothed by Gaussian smoothing.

### Signal synchronisation

The glove and sEMG records were synchronised by the acquisition software, especially designed for this purpose, to match the initial and final instants of each record. The acquisition software was programmed in C++ and glove and sEMG were synchronised by using the SDK libraries of the Cyberglove and Biometrics devices.

### Data resampling

The muscle activity recordings were resampled to 100 Hz to synchronise them with kinematic data.

### Data cutting and splitting

**Fig. 6:**
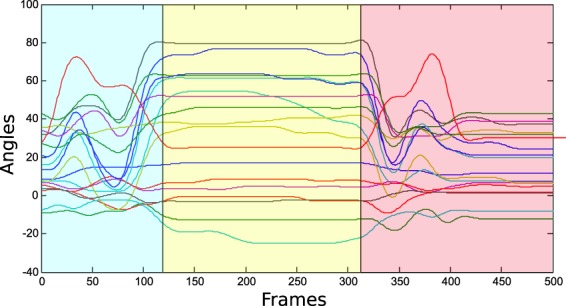
Example of label assignment. Each line corresponds to each joint measured (18 joints) for one subject during the performance of activity #5. Blue square corresponds with reaching, yellow square with manipulation and red square with releasing.

After synchronisation, the initial and final instants of each record (muscle activity and kinematics), during which the hands remained static, were trimmed. Records were then separated into the different phases (reaching, manipulation and release) by using the labelling performed by the operator while recording data. In some specific cases in which labelling data were missing, labelling was performed using the video recordings. In addition, a double check (manual label compared with expected kinematics from visual analysis) was performed in order to ensure the correct label assignment (Fig. 6).

## Data Records

### Data files

Data are presented as a single Matlab data structure (.*mat* file). This structure contains all the recorded kinematic and muscle activity data classified as: ADL, phase (reaching, manipulation or release) and subject. The data produced with the described methods were stored on Zenodo^19^. The fields contained in the structure are those detailed in the following scheme:

- Subject: subject ID;
- ADL: ADL ID, according to Table 1;
- Phase: phase of movement. 1 corresponds to reaching; 2 corresponds to manipulation; 3 corresponds to releasing;
- Time: Time stamp;
- Angles (18 columns): Calibrated anatomical angles in the following order (Table 2);

**Table 2:** Anatomical angles order (from top to bottom).

CMC1_A	Abduction of carpometacarpal 1
CMC1_F	Flexion of carpometacarpal 1
MCP1_F	Flexion of metacarpophalangeal 1
IP1_F	Flexion of interphalangeal 1
MCP2-3_A	Relative Abduction of metacarpophalangeal 2 and 3
MCP2_F	Flexion of metacarpophalangeal 2
PIP2_F	Flexion of proximal interphalangeal 2
MCP3_F	Flexion of metacarpophalangeal 3
PIP3_F	Flexion of proximal interphalangeal 3
MCP3-4_A	Relative Abduction of metacarpophalangeal 3 and 4
MCP4_F	Flexion of metacarpophalangeal 4
PIP4_F	Flexion of proximal interphalangeal 4
CMC5_F	Palmar Arch
MCP4-5_A	Relative Abduction of metacarpophalangeal 4 and 5
MCP5_F	Flexion of metacarpophalangeal 5
PIP5_F	Flexion of proximal interphalangeal 5
WRIST_F	Flexion of wrist
WRIST_A	Abduction of wrist

- Muscle activity (7 columns): Normalised sEMG signal for the seven representative spot areas (Fig. 3, ordered from Spot 1 to 7).

Raw sEMG data are also provided, without applying any filter and not resampled, so that researchers may choose to condition the signals as they please. The fields contained in this structure are those detailed in the following scheme:

- Subject: subject ID;
- ADL: ADL ID, according to Table 1;
- Time: Time stamp; this field corresponds with the time stamp of the previous structure;
- Raw EMG data (7 columns): Raw sEMG data, not filtered and not resampled, for the seven representative spot areas (Fig. 3, ordered from Spot 1 to 7);

### Sign criteria

**Fig. 7:**
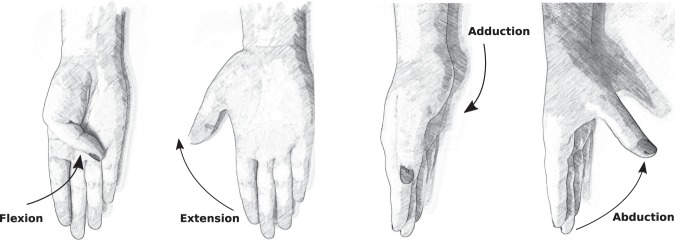
Sign criteria for the thumb CMC joint.

The sign criteria considered for the kinematics are shown in Table 3 and Fig. 7.

**Table 3:** Sign criteria considered.

PIP(2-5)_F, IP1_F, MCP(1-5)_F	Flexion+/Extension−
WRIST_F	Flexion+/Extension−
WRIST_A	Radial deviation+/Ulnar deviation−
MCP(2–3, 3–4, 4–5)_A	Fingers separated+/Fingers together−
P_Arch	Flexion+/Extension−
CMC1_F	Flexion+/Extension− (See Fig. 7)
CMC1_A	Abduction+/Adduction− (See Fig. 7)

## Technical Validation

### Data acquisition

All the recorded tasks were double checked (manual label compared with kinematic expected from visual analysis and, if it is the case, modified) to ensure that all the necessary labels to divide into elementary tasks were precisely identified.

In order to avoid any possible unexpected signal values, all the collected data were filtered using a second-order two-way low-pass Butterworth filter with a cut-off frequency of 5 Hz, as explained in previous sections.

### Experimental condition effect on hand kinematics

**Fig. 8:**
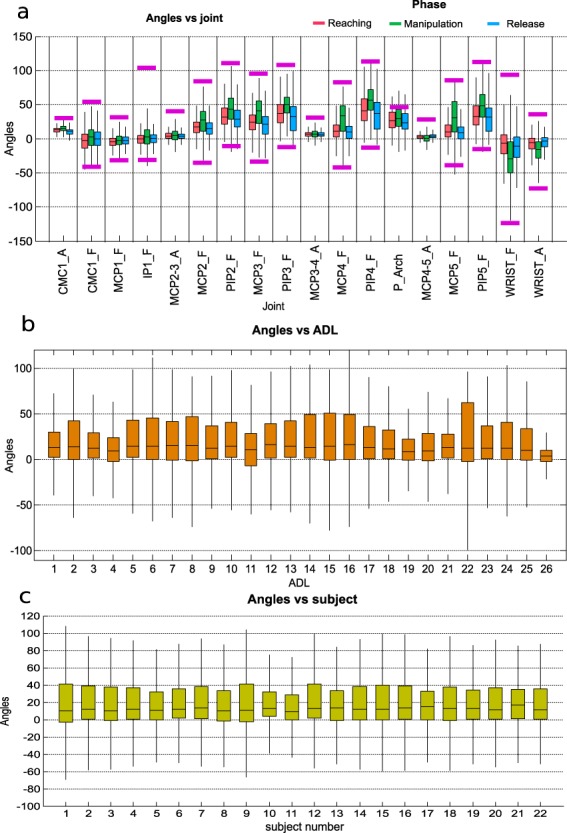
Effect of the experimental conditions on the hand kinematics. Subplots represent different experimental conditions: joints and phase (subplot **a**); ADL (subplot **b**), and subject (subplot **c**). The red horizontal central mark in the boxes is the median; the edges of the boxes are the 25th and 75th percentiles; whiskers extend to 1.5 times the interquartile range. AROMs for each joint are marked with pink lines.

In order to verify that data were similar to the data produced in real life, the effect of the experimental factors on the range of joint angles was evaluated. The factors that can affect joint angles are joint (as each one corresponds to a specific sensor), phase, subject or activity. The box and whisker graphs for all these factors are shown in Fig. 8. Active range of motion (AROMs) for each joint are marked, according to the literature^22,23^. Four ANOVAs were performed to the mean joint angles of the records, one for each factor (joint, phase, subjects and activities). Bonferroni adjustment was applied to compensate for multiple comparisons. For all the factors, the differences were significant (p < 0.05), although the post-hoc analysis to check the pairs with differences threw non homogeneous results: all the pairs of phases, almost all the pairs of joints, 50% of the pairs of activities and only 7% of the pairs of subjects were different.

A wide variability was noticed when considering how angles change in relation to joints. Joint angles have range values in accordance to active range of motion (AROM) values published in recent works^22,23^. Furthermore, all joint angles have, in general, range values within functional range of motion (FROM) values. In particular, the flexion of joints PIPs and MCPs presented a positive median with the largest FROM, which agrees with the literature^24^. The wrist flexion presented a negative median with a large FROM, and wrist deviation obtained a negative median value (ulnar deviation), but with a short FROM. This fact indicates that for most of the time, the wrist works in ulnar-extension during ADL, which agrees with previous studies^25^. When considering each phase, the manipulation phase gave the largest FROM, as expected: more complex joint movements are required during manipulation, resulting in larger FROM. Passive flexion/extension of joints while manipulating may also contribute to higher values of FROM. Reaching and release show similar values for all the joints, since movement during both phases is quasi symmetrical. When considering angles vs. ADL, all the activities generally obtained similar median and FROM values. In particular, activity 22 (Pouring water from the jug into the glass) presented the largest FROM, which agrees with the fact that this activity includes the flexion of all the fingers together with the wrist. Activity 26 (Cleaning the table with a cloth for 5 seconds) had the shortest FROM, which also agrees with the fact that this activity does not involve moving almost any hand joint as it only seems to require the elbow movement. We found no unusual data between subjects with similar median and ranges values.

### Experimental condition effect on muscle activity

**Fig. 9:**
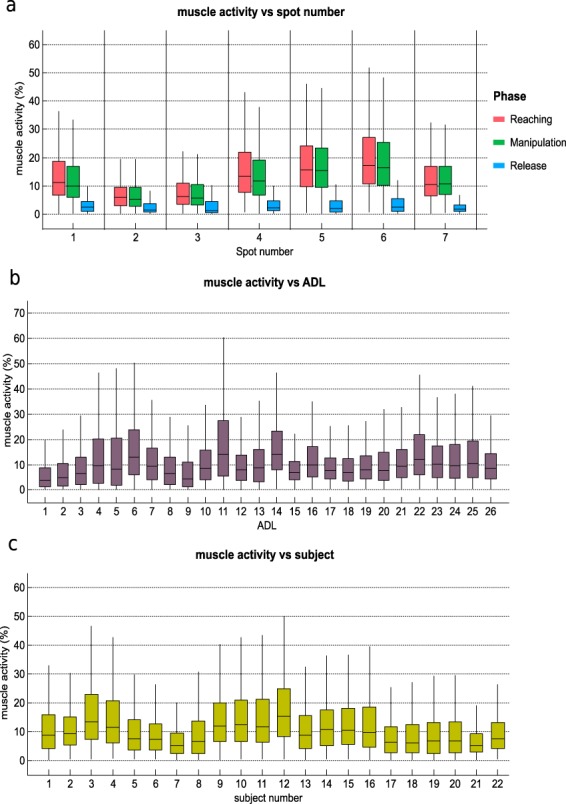
Effect of the experimental conditions on muscle activity. Subplots represent different experimental conditions: spots and phase (subplot **a**); ADL (subplot **b**); subject (subplot **c**). The horizontal central mark in the boxes is the median; the edges of the boxes are the 25th and 75th percentiles; whiskers extend to 1.5 times the interquartile range.

In order to verify that data were similar to those produced in real life, the effect of the experimental factors on muscle activity was evaluated. The factors that can affect muscle activity are spot number (as each corresponds to a specific location), phase, subject or activity. The box and whisker graphs were plotted for each factor and are shown in Fig. 9. Four ANOVAs were performed to the mean muscle activity of the records, one for each factor (spot number, phase, subjects and activities). Bonferroni adjustment was applied to compensate for multiple comparisons. For all the factors, the differences were significant (p < 0.05), although the post-hoc analysis to check the pairs with differences threw non homogeneous results: almost all the pairs of spots, 48% of the pairs of activities and 63% of the pairs of subjects were different. No significant differences were found between reaching and  manipulation phases.

When considering muscle activity vs. ADL, minor muscle activity was generally observed in all the ADL. This fact agrees with the fact that minimal muscle force is required to perform ADL^26^, as can be observed in Fig. 9, subplot A, where greater activity is shown during reaching (to place the hand to grasp) than during manipulation, as the tasks performed were low demanding. Reaching and manipulation phases had though higher muscle activity than release phase, as expected, because release is characterised by muscle relaxation. However, some ADL imply greater muscle activity values than others. This is acceptable if we consider that different ADL involve using distinct muscles, objects and force/wrench patterns (including variation of direction and sense) and, thus, lead to different muscle activity values. In particular, activity 11 (Unscrewing two lids and leaving them on the table) had the highest values, while activity 1 (Collecting a coin and placing it into a change purse) had the lowest values. Some variability was noted when considering muscle activity in relation to the recorded spot. In particular, spots 5 (finger extensors) and 6 (wrist extensors) presented the highest median values, which agrees with the fact that these muscles are the most active ones while performing ADL^26^. An agreement was also found with the kinematic data as the wrist seemed to work more in extension.

When considering muscle activity vs. subjects, some variability was observed among subjects. This is tolerable if we contemplate that some factors can affect muscle activity, such as different subjects being characterised by distinct anatomical characteristics, and the possibility of performing the same hand kinematics with different levels of effort according to the subject’s previous experience^27^. These facts highlight the different possibilities that each subject may have to carry out the same activities.

In conclusion, the KIN-MUS UJI dataset does not present any visible inappropriate effect that could prevent the database being used to improve current scientific advancements in robotics, rehabilitation, prosthetics, etc.

## Usage Notes

Many factors can affect the amplitude of the signal from sensors, including the acquisition setup, the subject’s anatomical characteristics, and fatigue, among others.

As wrist sensors do not well fit all hand sizes, an elastic band was used (Fig. 1) to achieve a better fit. However, sensor WRIST_F may provide more extreme values in extension due to the presence of cables underneath the globe. This effect is observed specially in ADL #7 and #11.

For ADL #25 of subject #22, all the data were lost. In this case, a Not a Number (NaN) value was presented.

Note that the sEMG records were normalised with the maximal values from any record (MVC and ADL). In particular, the maximum sEMG value on spot 4 was found in most subjects in ADL #11 (Unscrewing lids), but not during the MVCs records. This may suggest the usefulness of using similar MVC action to this activity in future works.

## Data Availability

The Matlab code used to calculate hand joint angles from CyberGlove instrumented gloves raw data can be accessed as open access^28^.
